# Multipose Binding in Molecular Docking

**DOI:** 10.3390/ijms15022622

**Published:** 2014-02-14

**Authors:** Kalina Atkovska, Sergey A. Samsonov, Maciej Paszkowski-Rogacz, M. Teresa Pisabarro

**Affiliations:** 1 Structural Bioinformatics, BIOTEC TU Dresden, Tatzberg 47-51, Dresden 01307, Germany; E-Mail: katkovs@gwdg.de; 2 Department of Medical Systems Biology, TU Dresden, Medical Faculty, University Hospital Carl Gustav Carus, Dresden 01307, Germany; E-Mail: maciej.paszkowski-rogacz@tu-dresden.de

**Keywords:** multipose binding, high-throughput docking, scoring optimization, binding affinity prediction

## Abstract

Molecular docking has been extensively applied in virtual screening of small molecule libraries for lead identification and optimization. A necessary prerequisite for successful differentiation between active and non-active ligands is the accurate prediction of their binding affinities in the complex by use of docking scoring functions. However, many studies have shown rather poor correlations between docking scores and experimental binding affinities. Our work aimed to improve this correlation by implementing a multipose binding concept in the docking scoring scheme. Multipose binding, *i.e.*, the property of certain protein-ligand complexes to exhibit different ligand binding modes, has been shown to occur in nature for a variety of molecules. We conducted a high-throughput docking study and implemented multipose binding in the scoring procedure by considering multiple docking solutions in binding affinity prediction. In general, improvement of the agreement between docking scores and experimental data was observed, and this was most pronounced in complexes with large and flexible ligands and high binding affinities. Further developments of the selection criteria for docking solutions for each individual complex are still necessary for a general utilization of the multipose binding concept for accurate binding affinity prediction by molecular docking.

## 1. Introduction

Continuous developments in technology and structural and functional genomics result in the discovery of many new proteins that can represent potential drug targets. This raises the necessity for new and fast computational approaches alternative to wet-lab high-throughput screens for drug discovery [1–3]. One of the most commonly used computational screening methods is molecular docking, which aims to predict the structure and binding affinity of a complex between a receptor and a ligand [3–5]. The docking procedure typically involves generating an ensemble of possible binding poses and their ranking by means of a certain scoring function [6–10]. In order to differentiate highly active ligands from weak or non-binders, proper scoring of the docked poses is required. Despite the proved success of molecular docking approaches [4,11–13], there are several comparative studies that show relatively poor correlation between docking scores and experimentally obtained binding affinities [14–17]. A recent study of seven commonly used docking programs applied to 1300 protein-ligand complexes showed a correlation in the range of 0.10–0.38 [16]. In another study, which evaluated the performance of nineteen scoring functions on experimentally solved crystal structures, the observed correlation of 0.35–0.76 indicates the current limitations of the scoring functions in predicting binding affinities [17]. One of the reasons for the rather weak performance of docking methods in these terms could be the fact that the used scoring schemes usually consider a single docking solution (pose). In this way, protein-ligand complexes that in nature exhibit several experimentally distinguishable binding modes are disregarded. In fact, experimentally solved structures of such complexes have already been reported [18–20].

In order to address multipose binding and its potential to improve the agreement between predicted and experimentally obtained data, we implemented the concept of multipose binding in the docking scoring scheme by taking into account several docking solutions for calculation of the binding affinity. Our aim was then to study the influence of multipose binding implementation on the binding affinity prediction by docking scoring functions in terms of several statistical measures for agreement between docking scores and experimental binding affinities. First, we investigated the ability of molecular docking and molecular dynamics (MD) simulations to identify multipose binding for four protein-ligand complexes, which were experimentally or computationally shown to exist in multiple binding modes. Then, a computational pipeline was established for high-throughput docking of three commonly used datasets of protein-small molecule complexes. For docking of each dataset, two docking programs, employing essentially different search algorithms and scoring functions, were used. For each analyzed complex a “single-pose” binding affinity was calculated by considering only one docking solution, *i.e.*, the one most similar to the experimentally solved structure. In addition, several “multipose” binding affinities were calculated by considering *n* docking solutions, including the pose used for the “single-pose” case. We determined the contribution of each selected pose score to the final binding energy, based on Boltzmann probabilities, as it has been previously successfully applied in binding energy calculations [21,22]. Taking several docking solutions into account could provide deeper insights into the structure and dynamics of a complex, and thus complement available structural data. Furthermore, such “multibinding” approach could be used to address cases in which the experimentally solved structure might not represent the energetically most favorable pose. Our work reveals general improvement of the agreement between the predicted binding affinities and experimental data when considering multiple docking solutions in comparison to the scores of the docking solutions most similar to the crystallographic complex. This improvement is found to be dependent on the ligand size and flexibility, as well as the binding affinity of the complex.

## 2. Results and Discussion

### 2.1. Computational Analysis of Complexes Shown to Exhibit Multipose Binding

We used AutoDock and MOE for molecular docking to identify multipose binding in three complexes already demonstrated to exhibit different binding modes in their respective experimentally solved structures (trypsin-inhibitor complex, HIV protease-inhibitor complex, and SH3 domain-polyproline peptide complex; see Methods Section for details). The corresponding binding poses were further studied by applying an MD approach and Mechanics/Poisson-Boltzmann Surface Area (MM-PBSA) free energy calculations. The annexin-heparin complex, though only shown to exhibit different orientations of binding computationally, was also included in this analysis since heparin is highly symmetrical in terms of its electrostatic properties and its binding to annexin is structurally dominated by simple electrostatic interactions, thus, making multipose binding more probable [23,24].

#### 2.1.1. Trypsin-Inhibitor Complex

**Figure 1.**
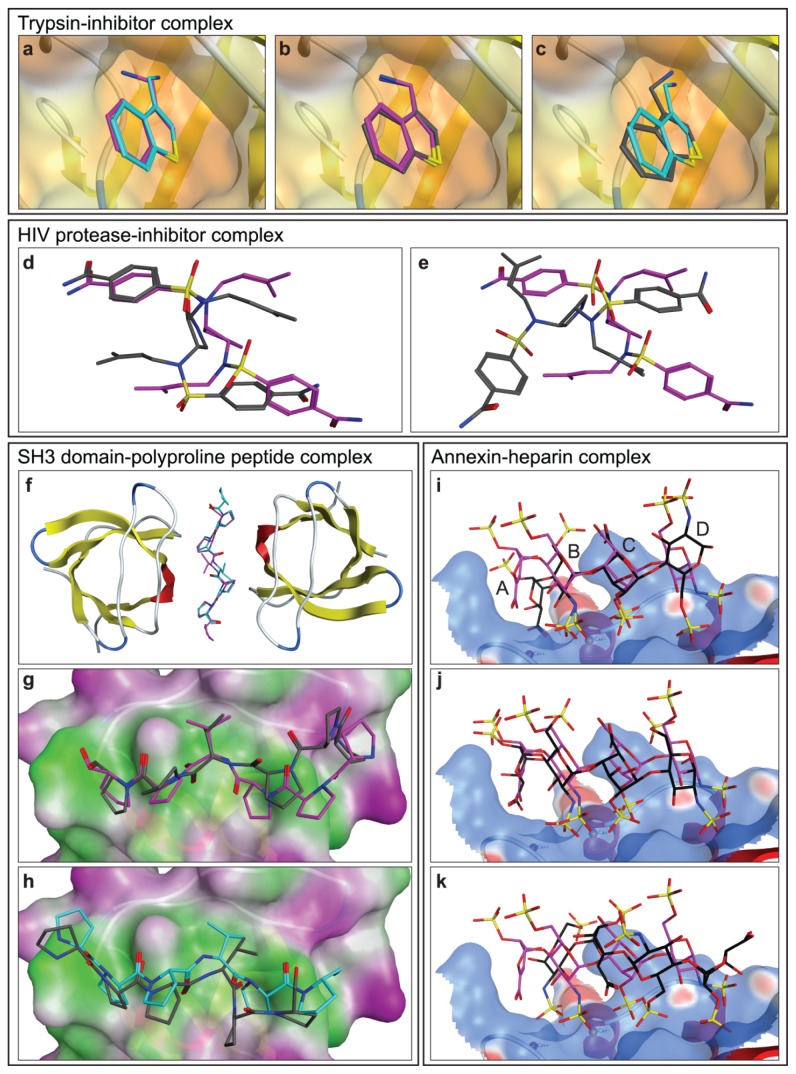
Complexes exhibiting multipose binding. (**a**,**b**,**c**) Trypsin-inhibitor complex. The protein receptor is depicted as a cartoon and an orange molecular surface. Crystal ligands in orientations I and II are shown in magenta and blue sticks, respectively. AutoDock docking solutions are shown in grey sticks; (**d**,**e**) HIV protease-inhibitor complex. The crystallographic ligand in orthorhombic orientation is shown in magenta sticks, and the AutoDock solutions analogous to orthorhombic (**d**) and to hexagonal (**e**) orientations are shown in grey sticks for comparison; (**f**,**g**,**h**) SH3 domain-polyproline peptide complex. The SH3 domain is depicted as a cartoon, and the crystal ligands in orientation I and II are shown in magenta and blue sticks, respectively. The crystal unit cell is shown in (**f**). In (**g**) and (**h**) a molecular surface is colored by lipophilicity (green: lipophilic, pink: hydrophilic), and the MOE docking solutions are shown in grey sticks (orientation I: (**g**), orientation II: (**h**)); and (**i**,**j**,**k**) Annexin A2-heparin complex. The protein is shown as a cartoon, and a molecular surface indicates the electrostatic potential (blue: positive, red: negative). The crystal ligand is shown in magenta sticks, and AutoDock docking solutions in black (poses I, II and III are shown in panels (**i**), (**j**) and (**k**), respectively). The sugar rings in the ligand are labeled for clarity (A, B, C and D).

The crystal structure of the complex of benzo [*b*] thiophene-3-ylmethanamine with trypsin shows two occupancies for the amine group of the ligand with respect to the protein receptor (orientation I and II; see Figure 1a). The poses adopted by the ligand in the two largest clusters generated by AutoDock were analogous to the two orientations observed in the crystal structure (Figure 1b,c). MOE also found both orientations of the ligand amine group within the two top solutions, in which orientation II observed in the crystal structure was ranked higher than orientation I. The MD analysis of the crystal structure revealed transition between two orientations, with orientation I prevailing during the simulation (~8.5 from 10 ns).

#### 2.1.2. HIV Protease-Inhibitor Complex

Two types of crystals (orthorhombic and hexagonal) corresponding to two distinguishable binding modes, named after the respective crystal form, were obtained in the structural characterization of the complex between HIV type I protease and 3,4-amino-pyrrolidine-based inhibitor [19]. The docking analysis of this complex was performed using the complex from the orthorhombic mode. AutoDock generated docking solutions analogous to both binding modes (Figure 1d,e), however they were not ranked among the top ten scored poses. The MD analysis of each of these solutions showed stability of the ligand in both orientations (RMSD of ligand heavy atoms converged at 1.7 and 3.2 Å for the hexagonal and orthorhombic poses, respectively). The top ranked docking solution obtained with MOE was analogous to the orthorhombic mode, while a solution analogous to the hexagonal mode was ranked at the 27th position.

#### 2.1.3. SH3 Domain-Polyproline Peptide Complex

SH3 domains have been computationally and experimentally shown to bind proline-rich motifs with moderate selectivity and specificity in two antiparallel orientations [25,26]. This ability of SH3 domains to bind ligands in two distinct binding modes has been extensively studied [25,27–30]. Gushchina *et al.* [20] reported the crystal structure of a chimeric protein constructed from a pseudo-wild type variant of the spectrin-SH3 domain and a proline-rich decapeptide connected to each other via a long linker. This structure reveals two different binding modes in the crystallographic unit cell, each consisting of a “triple” complex of two SH3 domains packed “face-to-face” and the PPPVPPY peptide ligand. In one of the binding modes the polyproline ligand is bound in orientation I with respect to one of the domains and in orientation II with respect to the other, while in the other binding mode the ligand has an opposite orientation (Figure 1f). In our docking study we used one SH3 domain complexed with the peptide ligand in orientation II. AutoDock yielded several clusters mainly representing the ligand in three orientations, which included both experimentally observed. The largest cluster was composed of poses analogous to orientation I. A second cluster, populated by poses characterized by binding energies comparable to the poses of the largest cluster, corresponded to orientation II. MOE also generated docking solutions analogous to both experimentally observed poses, with orientation II ranked higher than orientation I (Figure 1g,h). MD analysis of the crystal structure showed stable behavior of the ligand in orientation II (RMSDs of ligand heavy atoms converged at 1.5 Å), while the ligand in orientation I dissociated from the binding site after 7 ns. This might be reflective of the nature of the analyzed peptide, which was designed to bind preferentially in orientation II [20].

#### 2.1.4. Annexin A2-Heparin Complex

The formation of this complex is dominated by electrostatic interactions involving basic amino acid residues on the protein surface oriented in a way that matches the pattern of sulphate and carboxyl groups along the heparin chain. The symmetrical distribution of sulfate groups in heparin makes its sulfation pattern similar in both, parallel and antiparallel orientation with respect to the binding site, rendering these orientations physically possible as proposed computationally [24]. Docking analysis of this complex with AutoDock and MOE revealed three representative binding poses. In the most abundant one, the ligand adopted the same orientation as in the crystal structure but with a distinct conformation of the glycosidic linkage between the tetrasaccharide residues C and D, forcing the residue D to face the protein surface with the sulfate instead of the sulfoamino group (Figure 1i). The second representative pose was largely overlapping with the ligand from the crystal structure (Figure 1j). The third pose exhibited an antiparallel (opposite) orientation to the reference ligand (Figure 1k) in which the negatively charged groups facing the protein surface overlapped with the negatively charged groups from the crystallographic ligand structure, suggesting that the essential interactions for binding are preserved independently of the sugar backbone orientation. MD simulations showed stability for all three poses. The RMSD values for the ligand’s heavy atoms converged to 2.5, 3.5, and 3.0 Å for the first, second, and third pose, respectively.

In addition, we compared AutoDock scores and MD-based MM-PBSA free energies for the analyzed complexes (Table S1). Despite fundamental differences between these two approaches, such as treatment of flexibility and solvent, potential energy function and, in case of docking, challenges to score relatively big ligands (complexes 2–4), both estimate the different poses in complexes 1–3 as potential multipose binding. For complex 4, which is especially sensitive to electrostatic and solvent description due to the highly charged nature of heparin, pose II (in fact corresponding to the experimental structure) is clearly estimated to be the most favorable one by the MM-PBSA approach. This is not the case for AutoDock scoring. These results suggest that, in practice, when docking results point out a possibility for multipose binding, more accurate and rigorous MD-based analysis is further required.

To sum up, our results show that experimentally identified multiple poses can be computationally predicted by molecular docking and properly described by means of MD simulations. The observed challenge in terms of determining proper ranking of the binding poses matching the ones experimentally observed supports the idea that current scoring functions need to be improved. We further investigate whether considering more than one docking solution could assist in improving the scoring scheme.

### 2.2. High-Throughput Docking

Based on the described analysis of the complexes exhibiting multipose binding, and taking into account previous studies showing weak correlation between docking scores and experimentally obtained binding affinities, we aimed to investigate whether by considering several docking solutions for the scoring the agreement with experimental data could be improved. For this purpose, a high-throughput docking study was conducted on three datasets with eHiTS and AutoDock (see Methods Section for details). These programs were chosen among well-established docking methods since they are technically straightforward to automatize for high-throughput, and because they differ substantially in the search algorithm and the scoring function. AutoDock uses a genetic algorithm for generation of the docked poses and a semi-empirical force field-based scoring function, while eHiTS uses a fragment-based approach and a complex scoring function that combines a statistical and an empirical method. The calculations performed with these two methods and the three datasets yielded six sets of docking results, one for each dataset-docking program combination (DD-combination). Two docking solutions can be regarded as “standard” in a typical docking analysis, namely, the top score (the pose with the highest rank), and the best pose (the pose with lowest root mean square deviation (RMSD) to the reference ligand from the experimentally solved structure).

In general, eHiTS overperformed AutoDock in both, binding pose and binding energy prediction, for all DD-combinations. Table 1 shows the success rate of docking for each DD-combination, which is defined by assigning a favorable binding energy (Δ*G* < 0) to the best pose. According to this criterion, eHiTS docked successfully 99% of the complexes in all datasets. The reason for failure in most of the remaining 1% of complexes was the presence of a longer flexible linker between the rigid fragments than allowed by the program, and only in three cases from the Refined dataset (PDB IDs: 1UTC, 2Q8H, and 3DS9) and one complex from the CSAR dataset (PDB ID: 1IUP) no solution with favorable binding energy was generated. The docking procedure with AutoDock presented challenges at several steps. First, 50 complexes from the Refined dataset were removed as they contained non-standard metal ions in the receptor binding site (see Methods Section). Then, 106 complexes from the Refined set and seven complexes from the Core set were discarded due to various artifacts arising in the docking procedure (*i.e.*, size of the receptor exceeded the allowed limit; non-bonded atoms of various types were present; or no docking solution with favorable binding energy was generated). From the remaining complexes, 12 from the Core dataset and 229 from the Refined dataset did not score the best pose with favorable binding energy, resulting in 197 successfully docked complexes from the Core dataset (91%) and 2070 from the Refined dataset (84%). The least successful DD-combination was docking of the CSAR dataset with AutoDock. Here, a favorable binding energy was obtained for the best pose in only 65% of the cases. This unsatisfactory result might be due to the fact that the atomic partial charges from this dataset were inconsistent with the Gasteiger charges used by default in AutoDock, which is similar to previously reported observations [17]. Therefore, we excluded this DD-combination from further analysis.

The binding affinity prediction was assessed using the correlation between the predicted and experimental binding affinities (Table 2). We observed that eHiTS docking scores correlate substantially better with experimental data than AutoDock scores. The value of the Pearson correlation coefficient of the best pose scores to experimental data ranged from *r* ~ 0.07 to *r* ~ 0.58. Notably, the correlation of the top scores to experimental data, with exception of the CSAR-AutoDock combination, was always higher in comparison to best pose docking scores. The fraction of the complexes for which top scores corresponded to best poses ranged between 5% and 20% for different DD-combinations.

### 2.3. Implementation of the Multipose Binding Concept in Binding Affinity Prediction

The core objective of our work was to explore whether accounting for multiple poses in the docking scoring scheme could increase the agreement between predicted binding affinities and experimental data in comparison to a single-pose case, as is traditionally contemplated in docking. As previously discussed, the binding energy of the complex is normally calculated considering only one docking solution. In our work, we define as best pose the one closest to the crystal structure, which represents the only structure that can be claimed with sufficient certainty to occur in nature. Therefore, the scoring of the best pose was always taken as a baseline with option to be improved by considering multipose binding, and no other single-pose cases were compared with the multiple pose combinations.

The results from the analyzed multipose cases for the Refined dataset docked with eHiTS are shown in Table 3. Similarly, the same analysis was conducted for each of the remaining DD-combinations (Tables S2–S5). The most detailed analysis was conducted on the Refined-eHiTS combination since the general docking performance was the best, and because it was the DD-combination with the highest number of docked complexes, which could therefore yield the most statistically significant results.

#### 2.3.1. Refined-eHiTS

**Figure 2.**
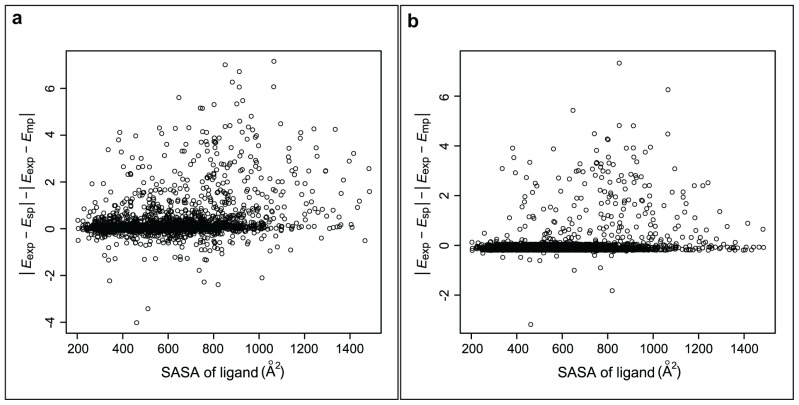
The difference of absolute single-pose and multipose residuals |*E*_exp_ − *E*_sp_| − |*E*_exp_ − *E*_mp_| is plotted against the solvent accessible surface area of the ligand. All values above zero denote an improvement of the binding affinity prediction. (**a**) Two-pose case considering the top score and the best pose from the Refined-eHiTS combination; and (**b**) Multipose case considering all poses from the Refined-eHiTS combination.

Docking with eHiTS yielded relatively good correlation of the single-pose binding affinities with experimental data (*r* = 0.47, Table 3). The relative improvement of the correlation observed in the BEST scenario was 21%, which defines the maximal possible effect that can be achieved by implementation of the multipose binding concept. This improvement is similar regardless of how many poses are considered in the multipose case, with the tendency of decrease of the improvement with increase of the number of poses. We also calculated the correlation between experimental data and a single-pose case constructed by “cherry picking” the poses with a predicted affinity closest to the experimental value. This can be used as another estimate of the upper limit of the possible improvement, and in such a case *r* = 0.63, which corresponds to a relative improvement of 34% (Table 3). Even in the RANDOM scenario, an improvement of the correlation was observed in all multipose cases, though not to the extent observed in the BEST scenario (*r* = 0.53, improvement ~13%). A similar trend was also noticed in the TOP scenario, in which lower number of poses yielded higher improvement. For example, considering two poses, the best pose and the top score, increased the correlation by 15%. This two-pose case is also illustrated in Figure 2a, which shows that the predicted binding affinity is not significantly affected for most of the complexes. Nevertheless, in those complexes, in which it is significantly affected, we observe mostly an improvement of its value, in a sense that it is closer to the experimental value than the binding affinity of a single pose. This gives a qualitative estimation on how big is the fraction of complexes, for which taking into account multipose binding in docking could be relevant and which, therefore, could represent multipose binding behavior. The lowest difference to the single-pose case was observed in the ALL scenario, with *r* = 0.52, resulting in an increase of the correlation by about 11%. When looking at the difference between absolute residuals in this scenario, the multipose binding affinity is closer to the experimental than the single-pose binding affinity in only 720 complexes, which accounts for less than one third of all complexes.

This leads to the conclusion that considering all docking solutions has a positive effect on the binding affinity prediction in only a limited number of cases (Figure 2b), which is to be expected since in most of the complexes only one binding pose is energetically significantly favorable over other ones. Nonetheless, the magnitude of this effect is sufficient to slightly improve the agreement with experimental data in terms of correlation and to achieve significant improvement in all cases in each scenario for the difference between the squared residuals analyzed with *t*-test (Table 3).

#### 2.3.2. Core-eHiTS and CASR-eHiTS

The statistical analysis of the docking results of the smaller datasets with eHiTS revealed similar observations as in the case of the Refined dataset (Tables S2 and S3). In particular, the improvement of the correlation with experimental data for the Core dataset was in a similar range, though slightly less than for the Refined (maximal increase by ~17% of the correlation for the BEST scenario, and by ~11% for the ALL scenario). The results obtained with the CSAR dataset showed higher correlation of the single-pose binding affinity to the experimental data (*r* = 0.58), thus, only a slight improvement of correlation was observed (*r* = 0.60 in average for the multipose cases). However, the effect of implementation of the multipose concept is clearly observed in the decrease of the sum of the squared residuals in comparison to the single-pose case (range of decrease 7%–21%). The *t*-test of the difference of the squared residuals for both small datasets did not revealed statistical significance, which might be due to the low number and high heterogeneity of complexes in these datasets.

#### 2.3.3. Refined-AutoDock and Core-AutoDock

The results obtained from AutoDock presented lower correlation to experimental data for the single-pose case in comparison to those obtained with eHiTS. Even so, an increase of the correlation coefficients can be observed in the TOP scenario for both datasets. The improvement of the agreement with experimental data is more clearly illustrated in the change of the sum of the squared residuals, which drops by 31% and 29% when two poses are considered in the BEST scenario, and by 23% and 19% when the best pose and the top score are considered, for the Refined and the Core dataset, respectively (Tables S4 and S5). The ALL scenario did not show decrease in the sum of squared residuals, implying no improvement of the values for the binding affinity. This means that only a limited number of additional poses could have practical use in the implementation of a multipose binding concept.

To summarize, regardless of the docking program and dataset used, the overall analysis implies that considering multiple docking solutions for binding affinity prediction with eHiTS or AutoDock scoring functions generally improves the agreement with experimental data in comparison to the scoring of the best pose docking solutions taken alone. When docking with eHiTS the improvement of the correlation ranged between 3.4% and 22.7%, and the decrease of the sum of the squared residuals ranged between 6.7% and 29.1%. When docking with AutoDock there was no significant improvement in terms of correlation coefficients, probably because the single-pose correlation is already rather poor. However, the decrease of the sum of the squared residuals clearly indicated the effect of implementation of the multipose binding concept, ranging up to 31%. The highest improvement of binding affinity prediction is in general observed in cases with a low number of poses. Due to the high heterogeneity of the datasets, this number could strongly vary for each individual complex, making this improvement even more dramatic in some cases.

However, we have to note that the observed improvement of the agreement with experimental data of the scores from the multipose binding scheme in comparison to the scores from the single-pose scheme was obtained by using very heterogeneous datasets and, therefore, could be attributed not only to multipose binding but also to inherent limitations of the docking scoring schemes. To estimate either impact represents a fair challenge within a high-throughput study. In contrast, for each individual complex obtained in high-throughput docking studies, which is suspected to represent multipose binding properties, more rigorous further analysis of dynamic stability and energetic characterization of the putative competing poses is required. When interpreting these data, particularly for the TOP scenario, it is important to keep in mind that due to the exponential nature of energy averaging, the impact of the top-ranked binding pose strongly prevails. Therefore, the multipose binding effect could be observed only in cases in which the best pose (which we always include) and the top-ranked pose (or several top poses) have close score values. For example, a difference of 1 kcal/mol between two poses defines their probabilities as 0.84 and 0.16. Most of the poses obtained by eHiTS for each complex differed in scores by about 1–1.5 kcal/mol, which allowed to take into account several poses with comparable weights.

### 2.4. Relation of the Physicochemical Properties of the Complex to the Observed Effect of Multipose Binding

The observed effect on the predicted binding affinity by implementation of the multipose binding concept in the scoring scheme is expressed to a different extent in different complexes. In order to investigate how this could be dependent on the physicochemical properties of the complex, we studied the relation of ten properties to the improvement of binding affinity prediction. The analysis was performed for two multipose cases, namely the two-pose case considering the top score and the best pose from the Refined-eHiTS and Refined-AutoDock combinations.

#### 2.4.1. Ligand Size

**Figure 3.**
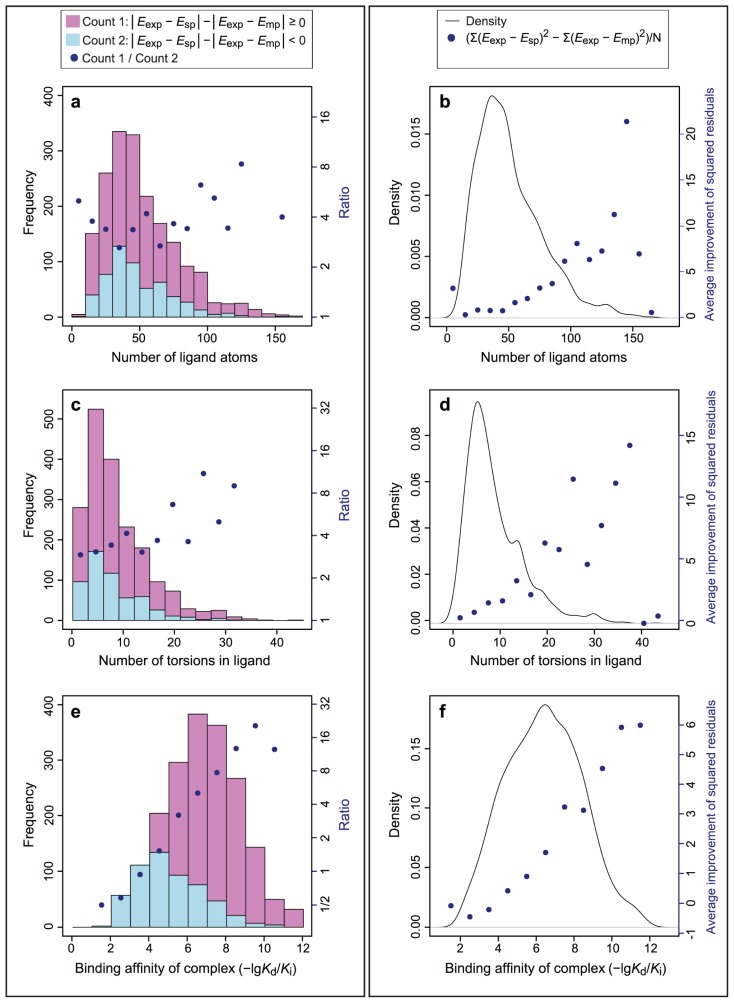
Analysis of the number of ligand atoms (**a**,**b**); ligand flexibility (**c**,**d**); and experimental binding affinity (**e**,**f**); in relation to the effect of multipose binding on binding affinity prediction for the two-pose case considering the top score and the best pose from the Refined-eHiTS combination. (**a**,**c**,**e**) Ratio of “improved” (pink) and “not-improved” (light blue) binding affinities; and (**b**,**d**,**f**) Average improvement of the squared residuals shown in dots and density of the ligand property shown as a line.

The ligand size was described using the number of atoms in the ligand, its molecular weight, its solvent accessible surface area and its molar refractivity, which carries information about the molecular volume [31]. The size of a ligand plays an important role in the docking process mainly due to two reasons. First, larger ligands generally present a greater docking challenge due to their, in principle, higher flexibility (*vide infra*). Second, larger ligands could possibly form higher number of interactions with the protein receptor in comparison to smaller ligands, thus giving rise to more possible combinations of these interactions and, consequently, more possible binding modes with comparable binding energies. Our data suggest that in the considered cases the positive effect tends to increase with the size of the ligand (Figures 3a,b, S1a,b and S2–S4). The calculated rank correlation coefficients support this observation (Table 4).

#### 2.4.2. Ligand Flexibility

Since more flexible ligands are expected to show higher probability of binding its receptor in different binding modes, it is important for ligand flexibility to be considered in terms of relation to the improvement of the binding affinity prediction due to multipose binding. Our results show an increase of the ratio between “improved” and “not-improved” binding affinities, as well as the average improvement of the squared residuals as the number of flexible torsions in the ligand increases (Figures 3c,d and S1c,d; Table 4). At the same time, this trend might be related to the intrinsic properties of the used docking programs. Namely, flexible ligands represent a higher challenge for docking, which results in poorer prediction of the binding poses that have wider range of binding affinities. Therefore, considering several poses for calculating the binding affinity can be expected to have a more pronounced effect.

#### 2.4.3. Binding Affinity

The most striking observation in this part of the study was that the improvement obtained for the prediction of the binding affinity by implementing the multipose binding concept tends to increase as the experimental binding affinity of the complex increases (Figures 3e,f and S1e,f; Table 4). This finding was not trivial, since the complexes with higher binding affinity were expected to show more specific binding and, therefore, to have less opportunities for multipose binding. The reason for this observation might partially originate from the nature of the docking programs, due to the fact that poses with low binding affinities are more challenging for computational identification. Additionally, Smith *et al.* [17] observe that a large portion of complexes where docking programs underestimate the binding affinity have high experimental affinities and *vice versa*. Taking that into account, as well as the fact that the considered two-pose case only improves the binding affinities of complexes where binding affinity was underestimated by the docking program, the highest improvement of the binding affinity prediction could be expected in complexes with high experimental binding affinities.

#### 2.4.4. Polar Properties

Two ligand features were considered as indicative of the polar properties of the ligand: the polar surface area (PSA) as an indicator of the ligand hydrophilicity, and log *P* as indicative of the lipophilicity of the ligand (Figures S5 and S6; Table 4). The hydrophilic properties play an important role in shaping the protein-ligand interaction by affecting the non-bonded contributions to the binding energy. PSA and log *P* may have an effect on the binding specificity and affinity and, consequently, an effect on the occurrence of multipose binding. In protein-small molecule complexes, polar interactions are normally accountable for the selectivity since they have both directional and distance requirements for optimal binding, while burying of apolar surface area is necessary for achieving high affinity [32]. Our results do not show any clear relation between the PSA and the effect of implementing the multipose binding concept. However, there is a slightly higher positive effect with increase of the log *P* value, possibly due to the ability of hydrophobic molecules to form less specific interactions. Furthermore, log *P* values of small molecules have been shown to correlate with their free energy of binding [33,34] suggesting that higher log *P* values correspond to higher binding affinities, which in turn correspond to higher positive effect on the binding affinity prediction by considering multipose binding.

#### 2.4.5. Ligand Charge and Number of Five- and Six-Membered Rings in the Ligand

For these two properties no conclusive assumption could be qualitatively made or based on the calculated correlation coefficients, which could be due to the high heterogeneity of the analyzed dataset (Figures S7 and S8; Table 4).

#### 2.4.6. PCA Analysis

In order to identify the ligand properties with highest influence on the observed effect on the binding affinity prediction, a principal component analysis for the difference between the squared residuals was performed for the two-pose case considering the top score and the best pose from the Refined-eHiTS combination. The inspection of the first two components, which account for 99.8% from the variance, suggested two properties to be the most influential, namely, the number of ligand atoms and its polar surface area (data not shown). As the average improvement of the squared residuals is expected to rise with the increase of these two properties, the PCA might imply that the subset of complexes with high number of atoms and high polar surface area can experience the greatest positive effect of employing the multipose binding concept for calculating binding affinities.

The performed analysis supports the notion that the extent of improvement of the binding affinity prediction as a result of considering multiple poses might depend on certain physicochemical properties of the complex. Larger and more flexible ligands are more susceptible to multipose binding. The highest dependence of the average improvement of the squared residuals from the analyzed properties in terms of the non-parametric correlation coefficients was observed for the experimental binding affinity, while the PCA analysis identified the number of ligand atoms and its polar surface area as most influential. The analysis of these properties can be used in the future for distinguishing complexes that could potentially benefit more from applying the multipose concept in docking scoring schemes.

## 3. Methods

### 3.1. Computational Analysis of Complexes Shown to Exhibit Multipose Binding

#### 3.1.1. Analyzed Structures

The following complexes were analyzed: trypsin-inhibitor complex (entry CC12313 from the DINGO dataset [35]), HIV protease type I-inhibitor complex (PDB IDs: 3CKT, 2ZGA), SH3 domain-polyproline peptide complex (PDB ID: 3THK) and annexin A2-heparin complex (PDB ID: 2HYU).

#### 3.1.2. AutoDock

The protein receptors and their ligands were pre-processed with AutoDockTools [36]. For docking experiments, the receptors were kept rigid, while maximal number of rotatable bonds was allowed for the ligands. All crystallographic water molecules were deleted from the initial structures, with the exception of the trypsin-inhibitor complex. Due to rather poor docking performance of this complex when discarding solvent, all water molecules within 4.5 Å distance from the ligand were retained. The atomic potential grid was calculated with 0.375 Å spacing in a box centered in the center of mass of the ligand and with distinct size for each complex (11.25 × 11.25 × 11.25 Å^3^ for the trypsin-inhibitor complex, 20.62 × 20.62 × 20.62 Å^3^ for the HIV protease-inhibitor complex, 18.75 × 18.75 × 21 Å^3^ for the SH3 domain-polyproline peptide complex and 22.5 × 22.5 × 24.37 Å^3^ for the annexin-heparin complex). Docking simulations were performed with Autodock4 using the genetic algorithm with default parameters and minimum of 2.5 × 10^6^ evaluations and 100 runs. Autodock3 was used for the annexin-heparin complex since, as previously observed, it overperforms Autodock4 for docking protein-glycosaminoglycan complexes [23,37].

#### 3.1.3. MOE Docking

Protein receptors and ligands were prepared with the default 3D protonation procedure in MOE [38]. Docking was performed using all default parameters with Triangle Matcher, retaining 300 poses and GBVI/WSA rescoring.

#### 3.1.4. MD Simulations

The MD simulations of the analyzed complexes were performed with AMBER 11 [39] using ff99SB force field parameters for the proteins, GLYCAM06 for heparin and gaff for the small molecules. The parameterization of the small molecules was performed by antechamber using the Gasteiger atomic charges. The used libraries for heparin were taken from our previous work [23]. Each complex was solvated in a truncated octahedron periodic box filled with TIP3P water molecules and neutralized by counterions. MD simulations were preceded by two energy-minimization steps of 10^3^ cycles of steepest descent and 500 cycles of conjugate gradient with harmonic force restraints on the protein receptor and ligand atoms, then 3 × 10^3^ cycles of steepest descent and 3 × 10^3^ cycles of conjugate gradient without constraints. This was followed by heating of the system to 300 K for 10 ps and a 50 ps MD equilibration run at 300 K and 10^5^ Pa in isothermal isobaric ensemble (NPT). Following the equilibration procedure, 10 ns of productive MD runs were carried out in NPT ensemble with Langevin temperature coupling with collision frequency parameter γ = 1 ps^−1^ and isotropic pressure scaling with pressure relaxation time of 2.0 ps. The SHAKE algorithm, 2 fs time integration step, 8 Å cutoff for non-bonded interactions and Particle Mesh Ewald method were used. In the case of the annexin/heparin complex NMR restraints for iduronic acid rings in ^1^C_4_ conformation were applied. The trajectory was analyzed with the ptraj module of AMBER. For binding free energy calculations, the MM-PBSA approach implemented in AMBER was used.

### 3.2. High-Throughput Docking

#### 3.2.1. Datasets

The two datasets used for high-throughput docking represent publicly available and manually curated collections of high-quality structural and binding affinity data for protein-small molecule complexes, widely used for molecular docking benchmarking and high-throughput studies [16,40–48]:

CSAR-NRC HiQ 2010 [49,50] (http://www.csardock.org/). This dataset contains 343 complexes and it was originally created for the purposes of the CSAR benchmark exercise of 2010 [17]. PDBbind 2011 [51,52] (http://www.pdbbind-cn.org). This database contains a collection of experimentally determined binding affinities representing protein–ligand complexes from the PDB. The PDBbind Refined subset contains 2455 complexes verified as high-quality structures, while the PDBbind Core subset, which contains 216 complexes, is selected from the Refined subset via a systematic non-redundant sampling process considering protein sequence similarity.

#### 3.2.2. Docking

The programs eHiTS [53] and AutoDock [36] were used for docking of the protein-small molecule complex datasets.

eHiTS 2009 [53,54] was used with default parameters. The ligand structure was used for the “clip” option reducing the receptor to only relevant parts to its binding site (within 10 Å distance of ligand). A representative subset of solutions was obtained using the default hierarchical clustering of the poses based on ligand atoms root mean square deviation (RMSD) and selecting the top scoring pose from each cluster.

AutoDock 4.2 [36]. The receptors and ligands were pre-processed with AutoDockTools (ADT) [36] using default parameters. All metal ions not recognized by AutoDock that were not located within 10 Å distance from the ligand were manually removed. All complexes containing such metal ions within 10 Å distance from the ligand were discarded from further studies. All protein receptors were treated rigid. All ligands were treated fully flexible in terms of torsional degrees of freedom. The atomic potential grid was calculated with 0.375 Å spacing in a box centered in the center of the ligand and with size twice as the ligand in each dimension. Docking simulations were performed with Autodock4 using genetic algorithm with default parameters and performing 3.0 × 10^6^ evaluations. For the relatively small ligand molecules contained in the used datasets, no significant changes were obtained when the number of runs varied from 50 to 100; therefore 50 runs were used in this case. The docking solutions were clustered based on RMSD of all ligand heavy atoms with clustering tolerance of 1.0 Å, and only the top scored poses of each cluster were considered for further calculations.

In both cases the available original atomic partial charges from the datasets (AM1-BCC for the ligands and AMBERparm94 for the receptors from the CSAR dataset and Gasteiger-Hueckel for the ligands from the PDBbind datasets) were retained in order to avoid any unnecessary pre-processing changes of the manually curated complexes. The missing partial charges of the receptors from the PDBbind dataset were calculated by the default procedures employed by each docking program, thus, optimizing docking performance.

### 3.3. Implementation of the Multipose Binding Concept in Binding Affinity Prediction

To introduce multipose binding in the docking scoring scheme, the binding energies Δ*G_i_* of several poses weighted by Boltzmann probabilities were considered:

(1) $$\Delta G=\sum_{i=1}^{N}p_{i}\Delta G_{i}=\frac{\sum_{i=1}^{N}e^{\frac{-\Delta \Delta G_{best,i}}{RT}}\Delta G_{i}}{\sum_{i=1}^{N}e^{\frac{-\Delta \Delta G_{best,i}}{RT}}}=\frac{\sum_{i=1}^{N}e^{\frac{-\Delta G_{i}}{RT}}\Delta G_{i}}{\sum_{i=1}^{N}e^{\frac{-\Delta G_{i}}{RT}}}$$

where Δ*G_best_* stands for the free binding energy of the pose with lowest heavy atoms RMSD to the crystal structure (best), and ΔΔ*G_best,i_* is the difference of the free binding energies of the pose *i* and the best pose. Docking solutions with Δ*G* ≥ 0 were discarded. For a direct comparison with the experimental data, the binding energies were converted to –p*K_d_* values. Different multipose cases were analyzed depending on the selection scheme of the used poses for calculation of the multipose binding affinity.

#### 3.3.1. Strategy for Pose Selection in Different Multipose Cases

The docking results from each dataset-docking program combination were analyzed in terms of 29 distinct multipose cases, grouped in four multipose scenarios: BEST, RANDOM, TOP and ALL. The pose with the lowest heavy-atoms RMSD value to the reference ligand from the complex crystal structure (best pose) was considered as the single-pose case, and it was always considered as one of the *n* selected poses.

The BEST scenario represents an artificial situation created with the purpose of discovering the upper limit of improvement of the binding affinity prediction that can be achieved. The selected poses were limited to only those that can improve the correlation to experimental data. For complexes in which the predicted values were lower (or higher) than the experimental, only poses with higher (or lower) binding affinities were taken as additional poses for calculating the multipose binding affinity, starting with the highest (or lowest) ranked pose. According to this criterion, multipose binding energies were calculated by taking into account the best pose and one to nine additional poses, as well as a case considering all better (or all worse) ranked poses, which resulted in a total of ten individual multipose cases. The RANDOM scenario represents a situation without bias towards the binding affinities of the selected poses. Specifically, multipose binding energies were calculated by taking into account the best pose and one to nine randomly selected poses from all docking solutions. The TOP scenario represents a situation in which the multipose binding affinity was calculated taking into account the best pose and one to nine top ranked poses. The ALL scenario was created with the purpose of removing any bias towards available structural or binding affinity data in the selection of the poses considered for the multipose case. Here, all docking solutions with favorable binding energy (Δ*G* < 0) were taken into account for calculation of the multipose binding affinity.

For illustrative purposes, an “upper-limit” single-pose case was constructed by selecting the docking solution closest to the respective experimental value for each complex.

#### 3.3.2. Statistical Analysis

The statistical analysis was performed with the statistical package R [55]. Due to the high heterogeneity of the datasets and in order not to be biased to a single statistical measure, several statistical measures were employed to assess the effect of considering multiple docking solutions on binding affinity prediction. The experimental binding affinity is denoted as *E*_exp_, the calculated single-pose binding affinity as *E*_sp_, and the calculated multipose binding affinity as *E*_mp_. The improvement of the correlation with experimental data was estimated with respect to the best pose as a single-pose case. First, the Pearson *r* and Spearman ρ correlation coefficients between *E*_exp_ and *E*_sp_, as well as *E*_exp_ and *E*_mp_ were calculated. Then, the squared residuals between the experimental and the calculated single-pose and multipose binding affinities were calculated, and Welch’s *t*-test was applied to detect the difference between the single-pose and multipose squared residuals. Further, the sums of all single-pose and multipose squared residuals were calculated and compared. Lastly, the difference of absolute residuals was considered, and the number of instances where the absolute single-pose residual was larger than the absolute multipose residual was calculated.

### 3.4. Relation of the Physicochemical Properties of the Complex to the Observed Effect of Multipose Binding

#### 3.4.1. Analyzed Properties

We analyzed the relation between the effect of implementing the multipose binding concept on binding affinity prediction, and the experimental binding affinity, as well as the relation with nine ligand properties (number of all atoms, molecular weight, solvent accessible surface area (SASA), molar refractivity, flexibility, polar surface area (PSA), lipophilicity expressed as a partition coefficient (log *P*), charge and number of five- and six-membered rings). The SASA of each ligand was determined by NACCESS [56] with values for the Bondi radii as defined in Amber 11 and a standard water molecule probe with 1.4 Å radius. The ligand flexibility was defined as number of torsional degrees of freedom in the ligand as determined by AutoDockTools. The PSA, the partition coefficient (log *P*) and the molar refractivity of the ligand were calculated by OpenBabel [57,58].

#### 3.4.2. Statistical Analysis

The effect of implementing the multipose binding concept on binding affinity prediction was illustrated by the following measures: (1) Ratio between “improved” and “not-improved” binding affinities, *i.e.*, the ratio between number of complexes in which multipose binding affinity value is closer (or farther) to the experimental value than their respective single-pose binding affinity; and (2) Average improvement of the squared residuals, *i.e.*, the mean of the difference of the single-pose and multipose squared residuals.

The relation of the analyzed properties to the described measures was primarily examined in a qualitative manner. Additionally, Kendall τ and Spearman ρ correlation coefficients between the difference of the squared residuals and each property were calculated. A principal component analysis (PCA) of the differences between the squared residuals was performed considering the following properties: the experimental binding affinity and six ligand properties (number of atoms, number of torsions, charge, PSA, log *P* and number of rings).

## 4. Conclusions

Molecular docking has been shown to be a useful tool in virtual screening of small molecules binding to proteins. However, there is still room for improvement of docking performance in terms of binding affinity prediction. We demonstrate that one of the reasons for the poor agreement between docking scores and experimental data might be the fact that docking methodologies neglect multipose binding, a phenomenon that has been experimentally observed. Our analysis of experimentally known complexes that exhibit multipose binding suggests that currently available computational methods are able to identify the observed multiple poses, and that the main challenge is rather their proper ranking, which stresses the need for improving docking scoring functions.

Our work represents an extensive docking study aiming to analyze the achievement of agreement between predicted and experimental binding affinities by considering multipose binding in the scoring schemes. Overall, taking multiple docking solutions into account for binding affinity prediction does have a positive effect in terms of improvement of the correlation with experimental data and decrease of the residuals between predicted and experimental binding affinities when compared to the single-pose binding affinity of the docking solution most similar to the reference crystal structure. The obtained relative improvement of up to 30% was exhibited for each dataset-docking program combination, with the most pronounced effect observed when a low number of poses was considered. The multipose case of considering the top scored and the best pose docking solutions produced solid improvement of the binding affinity prediction in comparison to the best pose case. Considering these two docking solutions produces on average a better prediction of the binding affinity to a different extent for different complexes, which might be due to two reasons: multipose binding or limitations of the docking scoring scheme. However, due to the highly heterogeneous nature of the used datasets and the fact that in most complexes multipose binding is not expected, averaging through all complexes might lead to underestimation of the observed effect. Therefore, multipose scoring should be carried out independently for each complex, using distinct number of poses to achieve the best agreement with the experiment. Larger and more flexible ligands show higher degree of improvement of the predicted binding affinity, maybe due to the greater potential to bind their respective receptors in multiple binding modes. For an individual complex, we could distinguish two scenarios: either the best pose (*i.e.*, experimental structure) is known, or it is not known. The presented high-throughput study and the obtained results clearly deal with the first case in order to learn whether adding another highly scored pose to the best pose might improve the scoring. When the best pose is not known, which, in practice, represents the majority of the cases, one should thoroughly analyze how close the scores of several top poses are. If they are similar, it might be a multipose binding case, in which these top poses should be further studied and compared by applying a more rigorous approach such as MD-based free energy calculation. However, if the scores of the obtained top poses are not similar, the exponential weights would essentially discard the influence of the lower scored poses on the estimated score of the complex, which at the end would correspond to the classical single-pose case.

The addition of multiple poses with exponential averaging presented in this study could also be seen as an implicit way of taking into account entropy in the docking calculations, which is usually overlooked by docking approaches since most of them account explicitly only for the entropic contribution related to the number of ligand’s torsional degrees of freedom.

To conclude, we show that considering multipose binding in docking might have a positive effect on the ability to better predict the binding affinity. Implementation of multipose binding in the scoring scheme yields a better assessment of the binding affinity of the analyzed complex to a different extent depending on the properties of the complex and the selection of the considered poses. Furthermore, exploration of multiple binding (*i.e.*, being able to distinguish the different contributions of each mode to the binding) may guide to a more efficient lead optimization process in rational design, as a proper understanding of the impact of each mode to the binding should be achieved prior to chemical modifications of a lead [59]. In summary, the effect of multipose binding on binding affinity prediction by docking scoring functions studied in this work shows that multipose binding should not be overlooked in the rational design process.

## Supplementary Information



## Figures and Tables

**Table 1.** Number of complexes (% from respective dataset in brackets) from each dataset/docking program combination for which results were successfully generated and the best pose was assigned favorable binding energy.

Dataset	eHiTS	AutoDock
**PDBbind Refined** (2455 complexes)	2428 (99%)	2070 (84%)
**PDBbind Core** (216 complexes)	214 (99%)	197 (91%)
**CSAR-NRC** (343 complexes)	340 (99%)	225 (65%)

**Table 2.** Pearson correlation between the predicted binding affinities of the “best pose” and the “top score” docking solutions and the experimental binding affinities for all dataset/docking program combinations.

Dataset/Program	Best pose	Top score	*N*_top = best_ (a), %
Refined/eHiTS	0.47	0.54	5.8
Core/eHiTS	0.46	0.51	8.4
CSAR/eHiTS	0.58	0.61	5.3
Refined/AutoDock	0.07	0.11	15.7
Core/AutoDock	0.14	0.18	15.2
CSAR/AutoDock	0.10	0.09	18.7

(a) Fraction of complexes, for which the top scores corresponded the best poses.

**Table 3.** Statistical analysis of the results obtained for the Refined-eHiTS combination.

Refined/eHiTS (2428 complexes)					
Number of poses	*r* (a)	ρ (b)	*p*-value (c)	∑(res_sp/mp_)^2^ (d)	Count (e)
**SINGLE-POSE**					
**1**	0.47	0.47	NA	16 651	NA
**BEST**					
**2**	0.57	0.56	2.76 × 10^−16^	11,810	2,117
**3**	0.57	0.56	1.42 × 10^−14^	12,083	2,066
**4**	0.56	0.55	2.66 × 10^−13^	12,287	2,023
**5**	0.56	0.55	1.46 × 10^−12^	12,417	1,989
**6**	0.56	0.55	6.55 × 10^−12^	12,535	1,966
**7**	0.56	0.55	1.93 × 10^−11^	12,623	1,933
**8**	0.56	0.55	4.79 × 10^−11^	12,698	1,910
**9**	0.56	0.55	1.07 × 10^−10^	12,766	1,878
**10**	0.55	0.54	2.22 × 10^−10^	12,829	1,858
**all better**(f)	0.53	0.52	1.06 × 10^−5^	13,929	1,603
**RANDOM**					
**2**	0.53	0.52	3.31 × 10^−7^	13,533	1,048
**3**	0.53	0.52	4.92 × 10^−7^	13,589	1,016
**4**	0.53	0.53	3.57 × 10^−7^	13,543	1,046
**5**	0.53	0.52	7.53 × 10^−7^	13,631	1,038
**6**	0.53	0.52	2.72 × 10^−7^	13,531	1,090
**7**	0.53	0.52	5.23 × 10^−7^	13,597	1,052
**8**	0.53	0.52	6.30 × 10^−7^	13,616	1,055
**9**	0.53	0.52	6.15 × 10^−7^	13,606	1,036
**10**	0.53	0.52	5.70 × 10^−7^	13,604	1,034
**TOP**					
**2**	0.54	0.53	7.33 × 10^−15^	12,055	1,784
**3**	0.54	0.53	4.17 × 10^−13^	12,349	1,625
**4**	0.54	0.53	7.51 × 10^−12^	12,563	1,527
**5**	0.54	0.53	4.59 × 10^−11^	12,711	1,441
**6**	0.54	0.53	2.25 × 10^−10^	12,845	1,376
**7**	0.54	0.53	7.80 × 10^−10^	12,955	1,312
**8**	0.54	0.53	2.13 × 10^−9^	13,046	1,263
**9**	0.54	0.53	5.47 × 10^−9^	13,134	1,214
**10**	0.53	0.52	1.23 × 10^−8^	13,212	1,158
**ALL**					
**all**	0.52	0.51	9.0 × 10^−4^	14,581	720
**“UPPER-LIMIT” SINGLE-POSE** **(g)**					
**1**	0.63	0.62	NA	11,115	NA

(a) Pearson correlation coefficient between *E*_exp_ and *E*_sp/mp_;

(b) Spearman rank-correlation coefficient between *E*_exp_ and *E*_sp/mp_;

(c) *p*-value from the (*E*_exp_ − *E*_sp_)^2^ *vs.* (*E*_exp_ − *E*_mp_)^2^ *t*-test;

(d) ∑(*E*_exp_ − *E*_sp/mp_)^2^, (kcal/mol)^2^;

(e) number of complexes where |*E*_exp_ − *E*_sp_| ≥ |*E*_exp_ − *E*_mp_|;

(f) a multipose case considering all poses with higher binding affinity than the single-pose, when the single-pose binding affinity is lower than the experimental, and all poses with lower binding affinity than the single-pose, when the single-pose binding affinity is higher than the experimental; and

(g) single-pose case constructed by selecting the poses with a score closest to the experimental affinity.

**Table 4.** Kendall τ and Spearman ρ rank-correlation coefficients between the difference of the squared residuals and the properties of the complex.

Property	Refined/eHiTS/TOP-BEST		Refined/AutoDock/TOP-BEST	

	ρ	τ	ρ	τ
Number of atoms	0.21	0.14	0.36	0.25
Molecular weight	0.22	0.15	0.34	0.23
Solvent accessible surface area	0.23	0.15	0.37	0.25
Molar refractivity	0.21	0.14	0.36	0.24
Flexible torsions	0.22	0.15	0.33	0.23
Binding affinity	0.48	0.33	0.44	0.30
Polar surface area	0.14	0.09	0.12	0.08
Log *P*	0.15	0.10	0.24	0.16
Number of rings	0.02	0.02	0.21	0.15
Charge	0.00	0.00	0.11	0.08

Values shown in italic were accompanied by *p*-value > 0.01.
