# Supplementary material for: Multipose Binding in Molecular Docking
Source: Int J Mol Sci. 2014 Feb 14;15(2):2622–45. doi: 10.3390/ijms15022622 (PMC3958872; doi:10.3390/ijms15022622)
Supplement: Supplementary file 1 [file ijms-15-02622-s001.pdf]

Supplementary Information

Table S1. Comparison of AD scores and MM-PBSA binding free energies for the analyzed complexes.

**Complex**	**Pose**	**AD scores, kcal/mol**	**$\Delta G_{MM\text{-}GBSA}$[a], kcal/mol**
1. Trypsin-inhibitor	Orientation I	−6.19	−15.7 ± 3.4
	Orientation II	−5.93	−20.2 ± 3.4
2. HIV protease-inhibitor	Orthorhombic	−5.09	−46.8 ± 6.3
	Hexagonal	−5.45	−56.0 ± 4.6
3. SH3 domain-polyproline	Orientation I	−4.71	−11.7 ± 3.8
	Orientation II	−4.39	−17.2 ± 4.3
4. Annexin A2-hepain	Pose I	−21.79	−38.4 ± 9.4
	Pose II	−19.15	−62.6 ± 8.8
	Pose III	−17.86	−42.1 ± 11.2

[a] The values are given with their standard deviations.

Table S2. Statistical analysis of the Core/eHiTS combination.

Core/eHiTS (214 complexes)					
**Number of poses**	r [(a)]	ρ [(b)]	p-value [(c)]	$\Sigma(\text{res}_{sp/mp})^{2}$ [(d)]	**Count** [(e)]
**SINGLE-POSE**					
**1**	0.46	0.45	NA	1746.1	NA
**BEST**					
**2**	0.54	0.52	0.07	1376.0	174
**3**	0.54	0.52	0.10	1401.0	168
**4**	0.54	0.52	0.12	1418.3	168
**5**	0.54	0.52	0.13	1429.3	165
**6**	0.54	0.52	0.14	1438.3	161
**7**	0.54	0.52	0.15	1444.0	160
**8**	0.54	0.52	0.16	1451.3	158
**9**	0.54	0.52	0.17	1458.8	157
**10**	0.54	0.52	0.18	1461.9	157
**all better**[(f)]	0.52	0.50	0.35	1544.3	131
**RANDOM**					
**2**	0.52	0.51	0.29	1518.6	83
**3**	0.51	0.50	0.35	1546.9	84
**4**	0.53	0.51	0.28	1515.6	87
**5**	0.51	0.49	0.29	1521.1	92
**6**	0.53	0.51	0.24	1498.9	97
**7**	0.52	0.50	0.32	1534.4	81
**8**	0.52	0.50	0.30	1523.1	83
**9**	0.51	0.49	0.31	1533.4	96
**10**	0.51	0.49	0.34	1543.7	88
**TOP**					
**2**	0.51	0.49	0.09	1398.4	145
**3**	0.51	0.49	0.12	1425.9	127
**4**	0.52	0.49	0.15	1442.9	126
**5**	0.52	0.49	0.17	1457.7	117
**6**	0.52	0.50	0.19	1469.0	112
**7**	0.52	0.50	0.20	1476.6	106
**8**	0.52	0.50	0.22	1484.8	103
**9**	0.52	0.50	0.23	1494.1	102
**10**	0.52	0.50	0.24	1498.1	100
**ALL**					
**all**	0.51	0.49	0.54	1612.8	60
**"UPPER-LIMIT" SINGLE-CASE** [(g)]					
**1**	0.61	0.59	NA	1294.2	NA

[(a)] Pearson correlation coefficient between E_{exp} and $E_{sp/mp}$; [(b)] Spearman rank-correlation coefficient between E_{exp} and $E_{sp/mp}$; [(c)] p-value from the $(E_{exp} - E_{sp})^2$ *vs.* $(E_{exp} - E_{mp})^2$ t-test; [(d)] $\Sigma(E_{exp} - E_{sp/mp})^2$, (kcal/mol)^2; [(e)] Number of complexes where $|E_{exp} - E_{sp}| \geq |E_{exp} - E_{mp}|$; [(f)] A multipose case considering all poses with higher binding affinity than the single-pose, when the single-pose binding affinity is lower than the experimental, and all poses with lower binding affinity than the single-pose, when the single-pose binding affinity is higher than the experimental; [(g)] single-pose case constructed by selecting the poses with a score closest to the experimental affinity.

Table S3. Statistical analysis of the CSAR/eHiTS combination.

CSAR/eHiTS (340 complexes)					
**Number of poses**	***r*** **[(a)]**	***ρ*** **[(b)]**	***p*-value [(c)]**	**$\Sigma(res_{sp/mp})^2$ [(d)]**	**Count [(e)]**
**SINGLE-POSE**					
**1**	0.58	0.57	NA	2194.5	NA
**BEST**					
**2**	0.64	0.62	0.03	1734.2	309
**3**	0.63	0.62	0.05	1774.8	301
**4**	0.63	0.61	0.07	1800.9	292
**5**	0.63	0.61	0.08	1816.0	291
**6**	0.63	0.61	0.10	1829.3	284
**7**	0.63	0.61	0.11	1840.1	279
**8**	0.63	0.61	0.12	1849.3	273
**9**	0.63	0.61	0.13	1857.7	269
**10**	0.62	0.61	0.14	1866.0	267
**all better[(f)]**	0.61	0.60	0.30	1961.5	222
**RANDOM**					
**2**	0.60	0.59	0.28	1955.2	153
**3**	0.60	0.58	0.31	1970.2	140
**4**	0.60	0.58	0.30	1963.1	138
**5**	0.60	0.58	0.34	1983.2	148
**6**	0.60	0.59	0.31	1968.8	147
**7**	0.60	0.59	0.27	1950.1	140
**8**	0.60	0.58	0.29	1959.2	148
**9**	0.61	0.60	0.24	1936.5	154
**10**	0.61	0.59	0.28	1956.6	149
**TOP**					
**2**	0.60	0.59	0.06	1780.6	264
**3**	0.61	0.59	0.09	1819.7	244
**4**	0.61	0.59	0.11	1847.0	224
**5**	0.61	0.59	0.13	1864.3	209
**6**	0.61	0.59	0.15	1878.6	199
**7**	0.61	0.59	0.17	1890.3	189
**8**	0.61	0.59	0.18	1900.7	176
**9**	0.61	0.59	0.20	1910.5	169
**10**	0.61	0.59	0.22	1920.9	165
**ALL**					
**All**	0.60	0.59	0.51	2048.2	105
**"UPPER-LIMIT" SINGLE-CASE [(g)]**					
**1**	0.68	0.66	NA	1630.1	NA

[(a)] Pearson correlation coefficient between E_{exp} and $E_{sp/mp}$; [(b)] Spearman rank-correlation coefficient between E_{exp} and $E_{sp/mp}$; [(c)] *p*-value from the $(E_{exp} - E_{sp})^2$ *vs.* $(E_{exp} - E_{mp})^2$ *t*-test; [(d)] $\Sigma(E_{exp} - E_{sp/mp})^2$, (kcal/mol)^2; [(e)] Number of complexes where $|E_{exp} - E_{sp}| \geq |E_{exp} - E_{mp}|$; [(f)] A multipose case considering all poses with higher binding affinity than the single-pose, when the single-pose binding affinity is lower than the experimental, and all poses with lower binding affinity than the single-pose, when the single-pose binding affinity is higher than the experimental; [(g)] single-pose case constructed by selecting the poses with a score closest to the experimental affinity.

Table S4. Statistical analysis of the Refined/AutoDock combination.

Refined/AutoDock (2070 complexes)					
**Number of poses**	***r*** **(a)**	***ρ*** **(b)**	***p*-value (c)**	**$\Sigma(res_{sp/mp})^{2}$ (d)**	**Count (e)**
			**SINGLE-POSE**		
**1**	0.07	0.09	NA	22,285	NA
			**BEST**		
**2**	0.18	0.22	1.80×10^{-12}	15,380	1,488
**3**	0.15	0.19	8.14×10^{-8}	16,923	1,509
**4**	0.14	0.17	7.50×10^{-6}	17,760	1,515
**5**	0.13	0.16	1.04×10^{-4}	18,334	1,516
**6**	0.12	0.15	5.26×10^{-4}	18,737	1,520
**7**	0.12	0.14	0.00162	19,045	1,519
**8**	0.12	0.14	0.00420	19,330	1,516
**9**	0.11	0.14	0.00872	19,567	1,511
**10**	0.11	0.13	0.01484	19,753	1,509
**all better (f)**	0.09	0.11	0.46593	21,500	1,472
			**RANDOM**		
**2**	0.08	0.10	0.5688	21,672	697
**3**	0.07	0.09	0.5099	21,578	689
**4**	0.08	0.10	0.5567	21,657	652
**5**	0.08	0.10	0.4602	21,496	653
**6**	0.08	0.10	0.5241	21,606	584
**7**	0.08	0.10	0.6205	21,752	565
**8**	0.08	0.10	0.5090	21,579	556
**9**	0.07	0.10	0.6094	21,735	546
**10**	0.07	0.09	0.5629	21,663	544
			**TOP**		
**2**	0.11	0.15	4.60×10^{-7}	17,169	1,286
**3**	0.10	0.13	0.00018	18,433	1,231
**4**	0.10	0.13	0.00231	19,138	1,155
**5**	0.09	0.12	0.01189	19,667	1,104
**6**	0.09	0.12	0.03058	20,027	1,049
**7**	0.09	0.11	0.06098	20,320	991
**8**	0.09	0.11	0.10038	20,558	947
**9**	0.08	0.11	0.15154	20,773	901
**10**	0.08	0.10	0.20691	20,951	861
			**ALL**		
**All**	0.07	0.09	0.8220	22,531	379
			**"UPPER-LIMIT" SINGLE-CASE (g)**		
**1**	0.35	0.39	NA	12,678	NA

(a) Pearson correlation coefficient between E_{exp} and $E_{sp/mp}$; (b) Spearman rank-correlation coefficient between E_{exp} and $E_{sp/mp}$; (c) *p*-value from the $(E_{exp} - E_{sp})^2$ *vs.* $(E_{exp} - E_{mp})^2$ *t*-test; (d) $\Sigma(E_{exp} - E_{sp/mp})^2$, $(kcal/mol)^2$; (e) Number of complexes where $|E_{exp} - E_{sp}| \geq |E_{exp} - E_{mp}|$; (f) A multipose case considering all poses with higher binding affinity than the single-pose, when the single-pose binding affinity is lower than the experimental, and all poses with lower binding affinity than the single-pose, when the single-pose binding affinity is higher than the experimental; (g) single-pose case constructed by selecting the poses with a score closest to the experimental affinity.

Table S5. Statistical analysis of the Core/AutoDock combination.

Core/AutoDock (197 complexes)					
**Number of poses**	***r*** [(a)]	***ρ*** [(b)]	***p*-value** [(c)]	**Σ(res$_{sp/mp}$)^{2}** [(d)]	**Count** [(e)]
			**SINGLE-POSE**		
**1**	0.14	0.15	NA	2062.4	NA
			**BEST**		
**2**	0.28	0.32	0.05	1454.6	138
**3**	0.24	0.28	0.13	1586.7	140
**4**	0.22	0.25	0.20	1656.0	143
**5**	0.21	0.23	0.28	1721.1	144
**6**	0.20	0.22	0.34	1762.2	143
**7**	0.20	0.21	0.39	1788.6	142
**8**	0.19	0.21	0.43	1810.4	141
**9**	0.19	0.20	0.46	1828.1	140
**10**	0.18	0.19	0.52	1857.5	140
**all better**[(f)]	0.16	0.17	0.83	1990.3	137
			**RANDOM**		
**2**	0.13	0.15	0.88	2014.4	68
**3**	0.15	0.16	0.80	1981.3	61
**4**	0.13	0.14	0.87	2116.1	56
**5**	0.13	0.14	0.93	2033.9	52
**6**	0.15	0.16	0.87	2008.7	59
**7**	0.13	0.15	0.92	2028.2	48
**8**	0.14	0.16	0.98	2053.4	51
**9**	0.14	0.14	0.98	2056.0	47
**10**	0.15	0.16	0.89	2018.2	48
			**TOP**		
**2**	0.18	0.23	0.22	1674.4	113
**3**	0.18	0.21	0.35	1767.3	106
**4**	0.17	0.19	0.48	1836.1	103
**5**	0.16	0.19	0.58	1882.7	97
**6**	0.16	0.18	0.65	1916.4	88
**7**	0.16	0.18	0.69	1934.2	83
**8**	0.16	0.17	0.73	1951.7	77
**9**	0.16	0.17	0.75	1960.1	78
**10**	0.15	0.16	0.82	1986.7	78
			**ALL**		
**all**	0.14	0.15	0.97	2075.6	41
			**"UPPER-LIMIT" SINGLE-CASE** [(g)]		
**1**	0.42	0.47	NA	1289.8	NA

[(a)] Pearson correlation coefficient between E_{exp} and $E_{sp/mp}$; [(b)] Spearman rank-correlation coefficient between E_{exp} and $E_{sp/mp}$; [(c)] *p*-value from the $(E_{exp} - E_{sp})^2$ *vs.* $(E_{exp} - E_{mp})^2$ *t*-test; [(d)] $\Sigma(E_{exp} - E_{sp/mp})^2$, (kcal/mol)^2; [(e)] Number of complexes where $|E_{exp} - E_{sp}| \geq |E_{exp} - E_{mp}|$; [(f)] A multipose case considering all poses with higher binding affinity than the single-pose, when the single-pose binding affinity is lower than the experimental, and all poses with lower binding affinity than the single-pose, when the single-pose binding affinity is higher than the experimental; [(g)] single-pose case constructed by selecting the poses with a score closest to the experimental affinity.

Figure S1. Analysis of the number of ligand atoms (**a**,**b**), ligand flexibility (**c**,**d**) and experimental binding affinity (**e**,**f**) in relation to the effect of multipose binding on binding affinity prediction for the two-pose case considering the top score and the best pose from the Refined-AutoDock combination. (**a**,**c**,**e**) Ratio of "improved" (pink) and "not-improved" (light blue) binding affinities; and (**b**,**d**,**f**) Average improvement of the squared residuals shown in dots and density of the ligand property shown as a line.

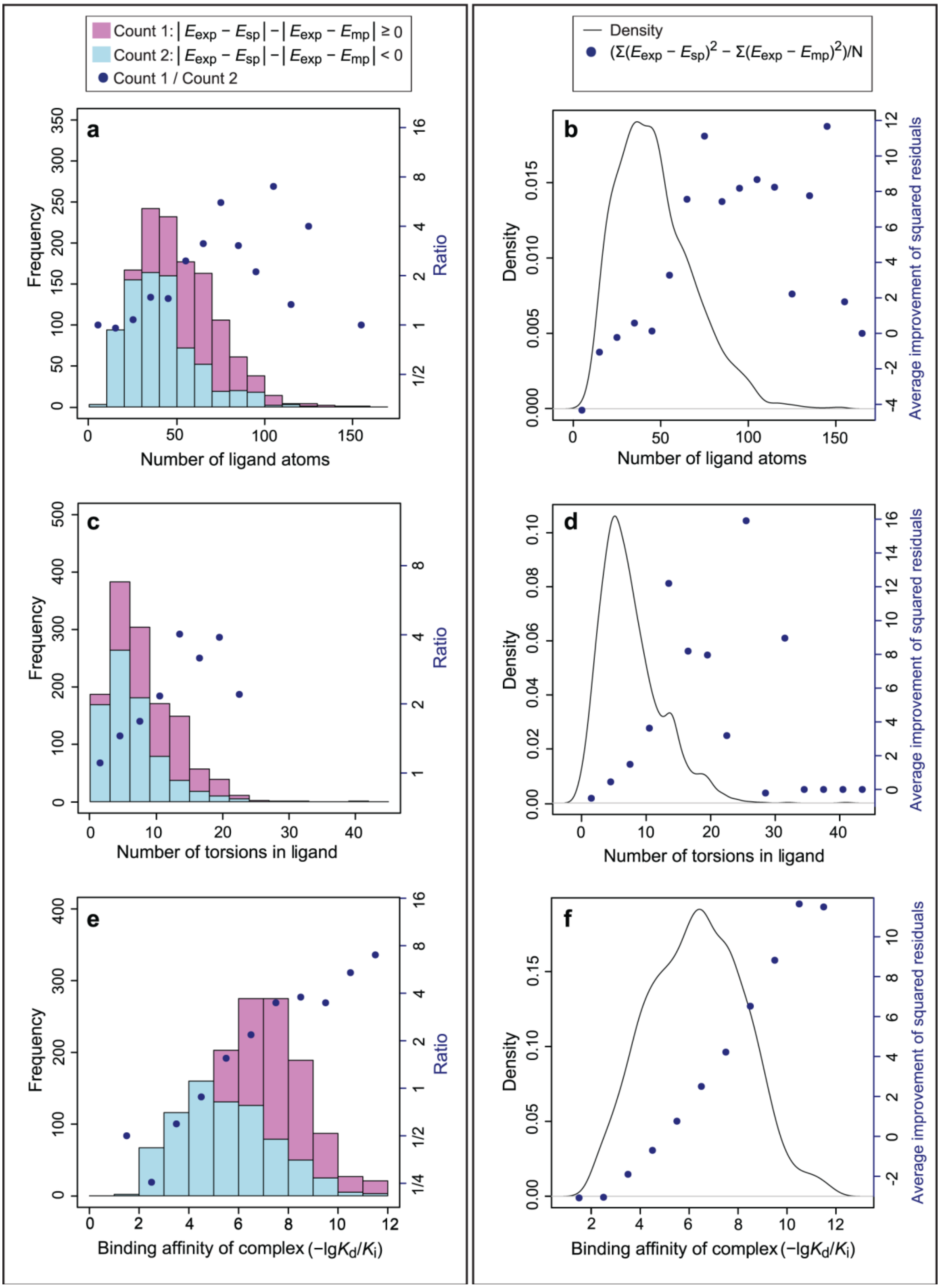

Figure S2. Analysis of the molecular weight of the ligands in relation to the effect of multipose binding on binding affinity prediction for the two-pose case considering the top score and the best pose from the Refined-eHiTS (**a**,**b**) and the Refined-AutoDock combination (**c**,**d**). (**a**,**c**) Ratio of "improved" (pink) and "not-improved" (light blue) binding affinities; and (**b**,**d**) Average improvement of the squared residuals shown in dots and density of the ligand property shown as a line.

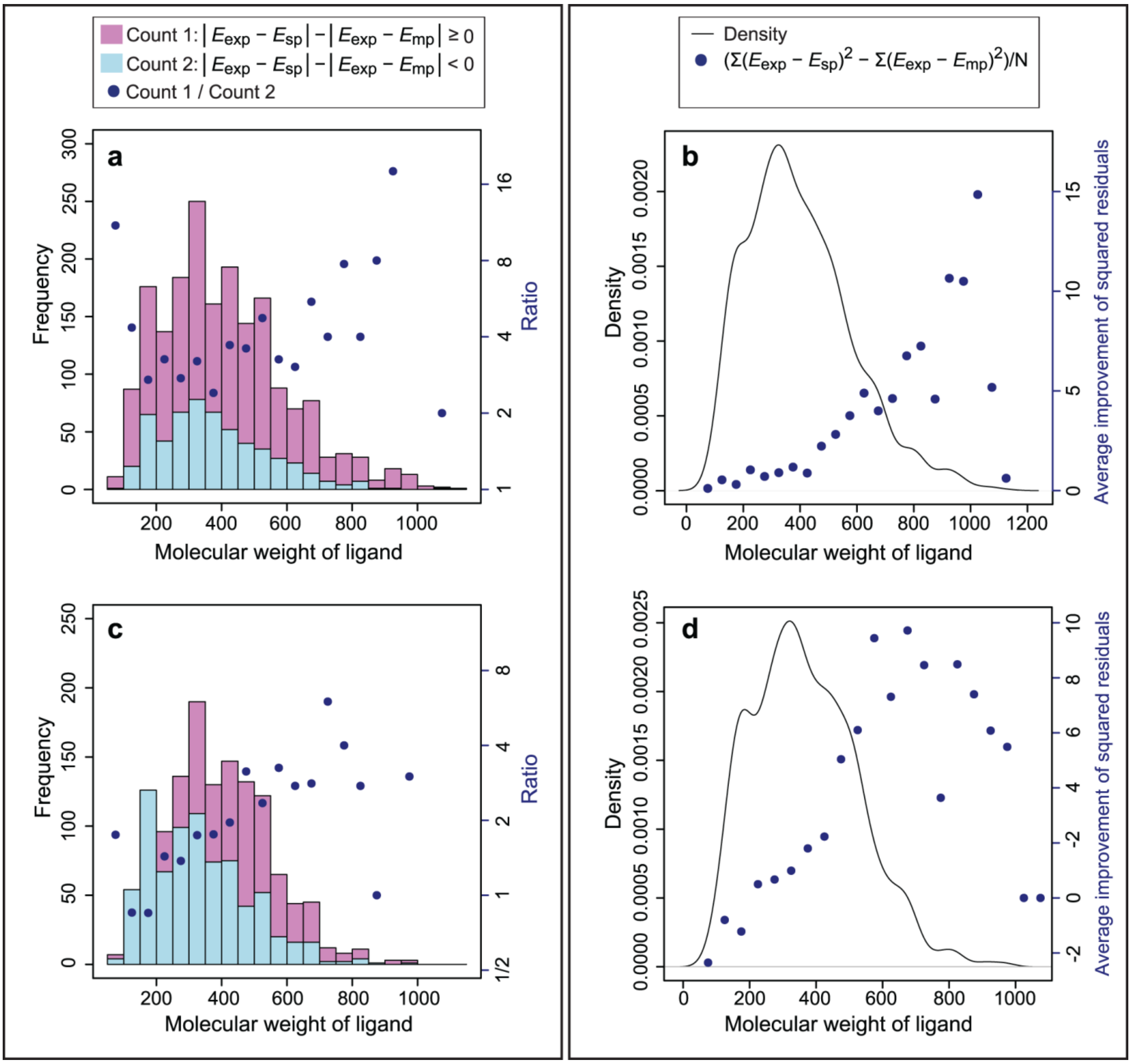

Figure S3. Analysis of the solvent accessible surface area of the ligands in relation to the effect of multipose binding on binding affinity prediction for the two-pose case considering the top score and the best pose from the Refined-eHiTS (**a**,**b**) and the Refined-AutoDock combination (**c**,**d**). (**a**,**c**) Ratio of "improved" (pink) and "not-improved" (light blue) binding affinities; (**b**,**d**) Average improvement of the squared residuals shown in dots and density of the ligand property shown as a line.

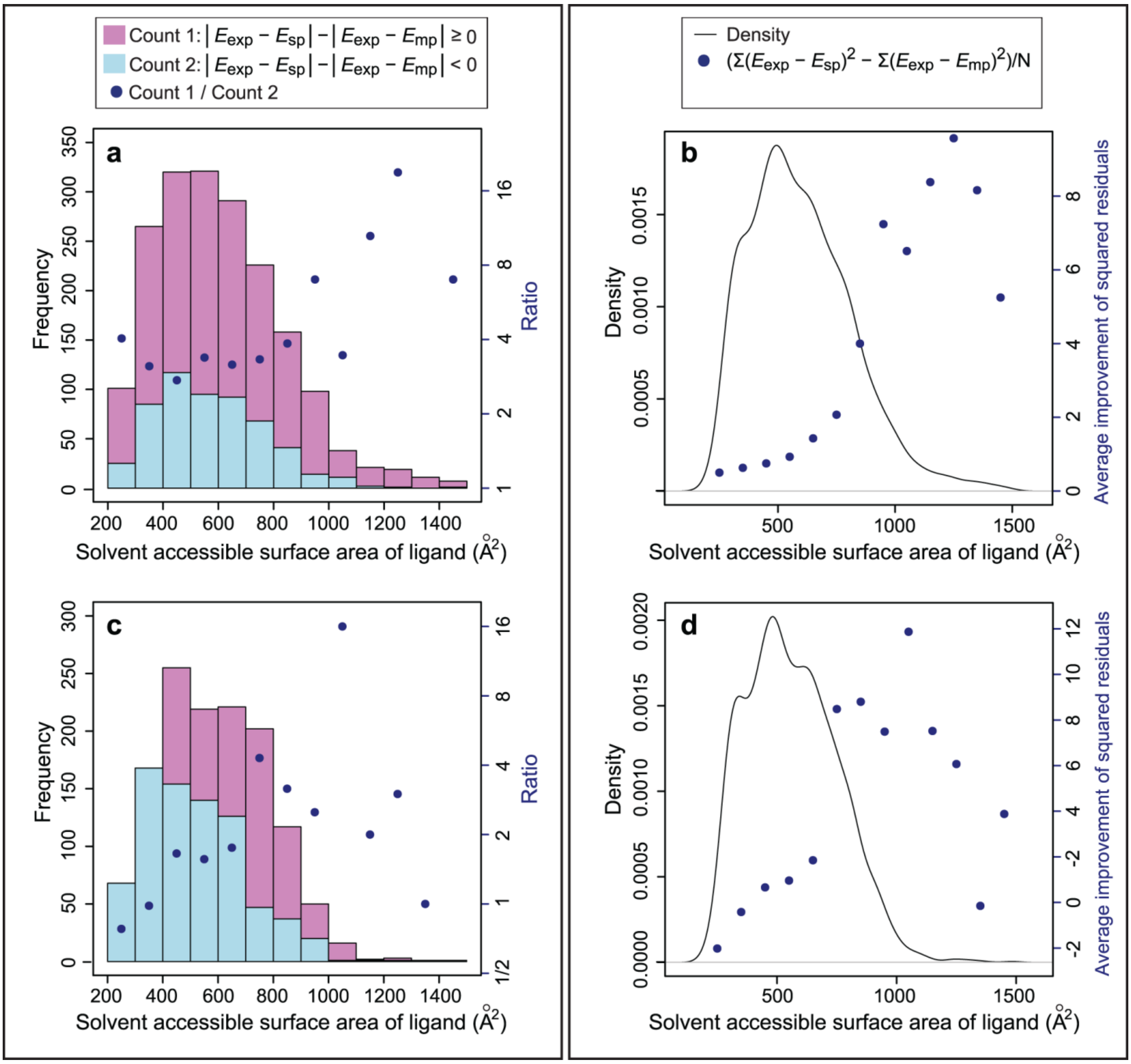

Figure S4. Analysis of the molar refractivity of the ligands in relation to the effect of multipose binding on binding affinity prediction for the two-pose case considering the top score and the best pose from the Refined-eHiTS (**a**,**b**) and the Refined-AutoDock combination (**c**,**d**). (**a**,**c**) Ratio of "improved" (pink) and "not-improved" (light blue) binding affinities; and (**b**,**d**) Average improvement of the squared residuals shown in dots and density of the ligand property shown as a line.

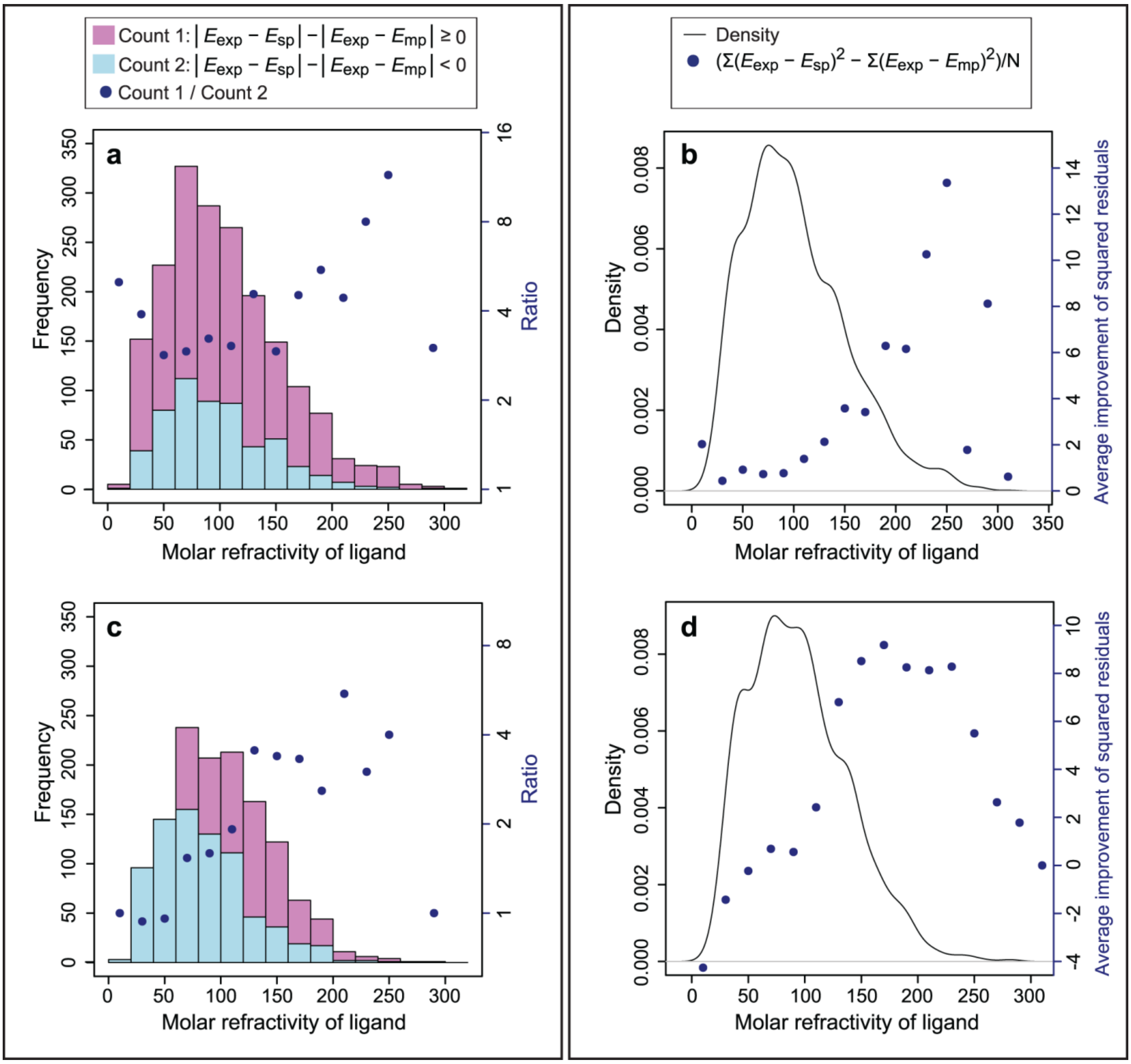

Figure S5. Analysis of the polar surface area of the ligands in relation to the effect of multipose binding on binding affinity prediction for the two-pose case considering the top score and the best pose from the Refined-eHiTS (**a**,**b**) and the Refined-AutoDock combination (**c**,**d**). (**a**,**c**) Ratio of "improved" (pink) and "not-improved" (light blue) binding affinities; and (**b**,**d**) Average improvement of the squared residuals shown in dots and density of the ligand property shown as a line.

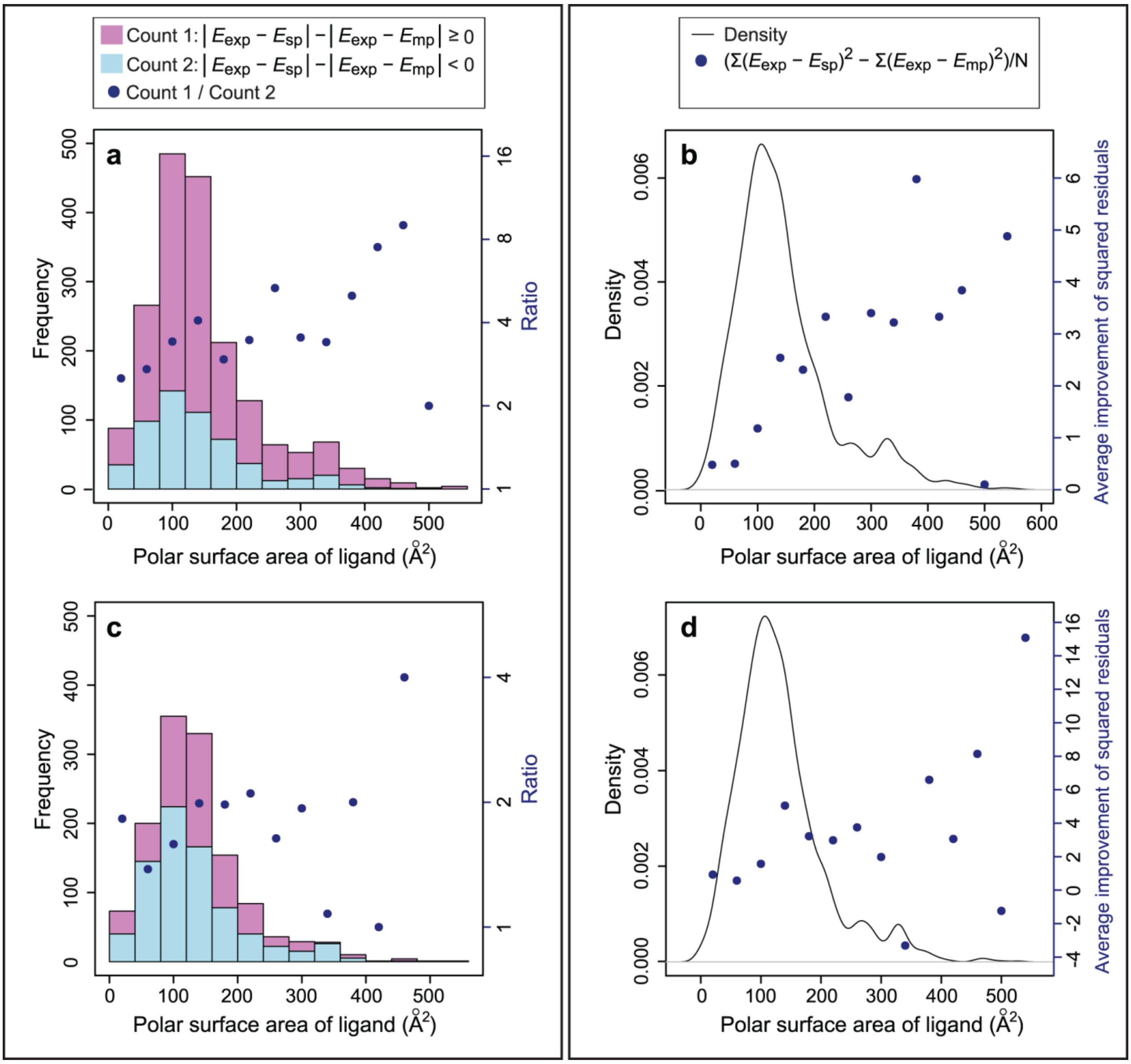

Figure S6. Analysis of the logP of the ligands in relation to the effect of multipose binding on binding affinity prediction for the two-pose case considering the top score and the best pose from the Refined-eHiTS (**a**,**b**) and the Refined-AutoDock combination (**c**,**d**). (**a**,**c**) Ratio of "improved" (pink) and "not-improved" (light blue) binding affinities; and (**b**,**d**) Average improvement of the squared residuals shown in dots and density of the ligand property shown as a line.

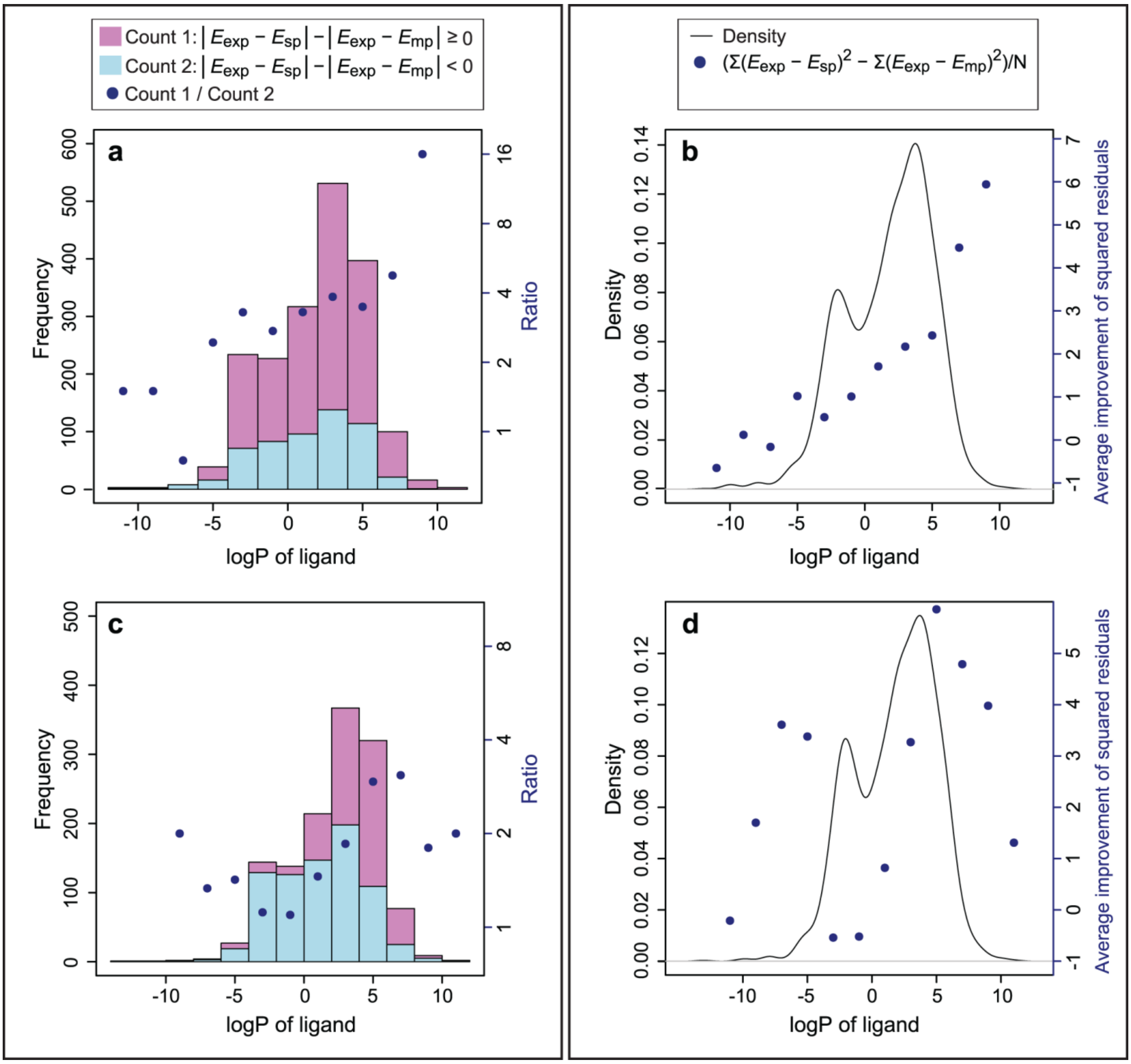

Figure S7. Analysis of the number of rings in the ligand in relation to the effect of multipose binding on binding affinity prediction for the two-pose case considering the top score and the best pose from the Refined-eHiTS (**a**,**b**) and the Refined-AutoDock combination (**c**,**d**). (**a**,**c**) Ratio of "improved" (pink) and "not-improved" (light blue) binding affinities; and (**b**,**d**) Histogram of the ligand property and average improvement of the squared residuals shown in dots.

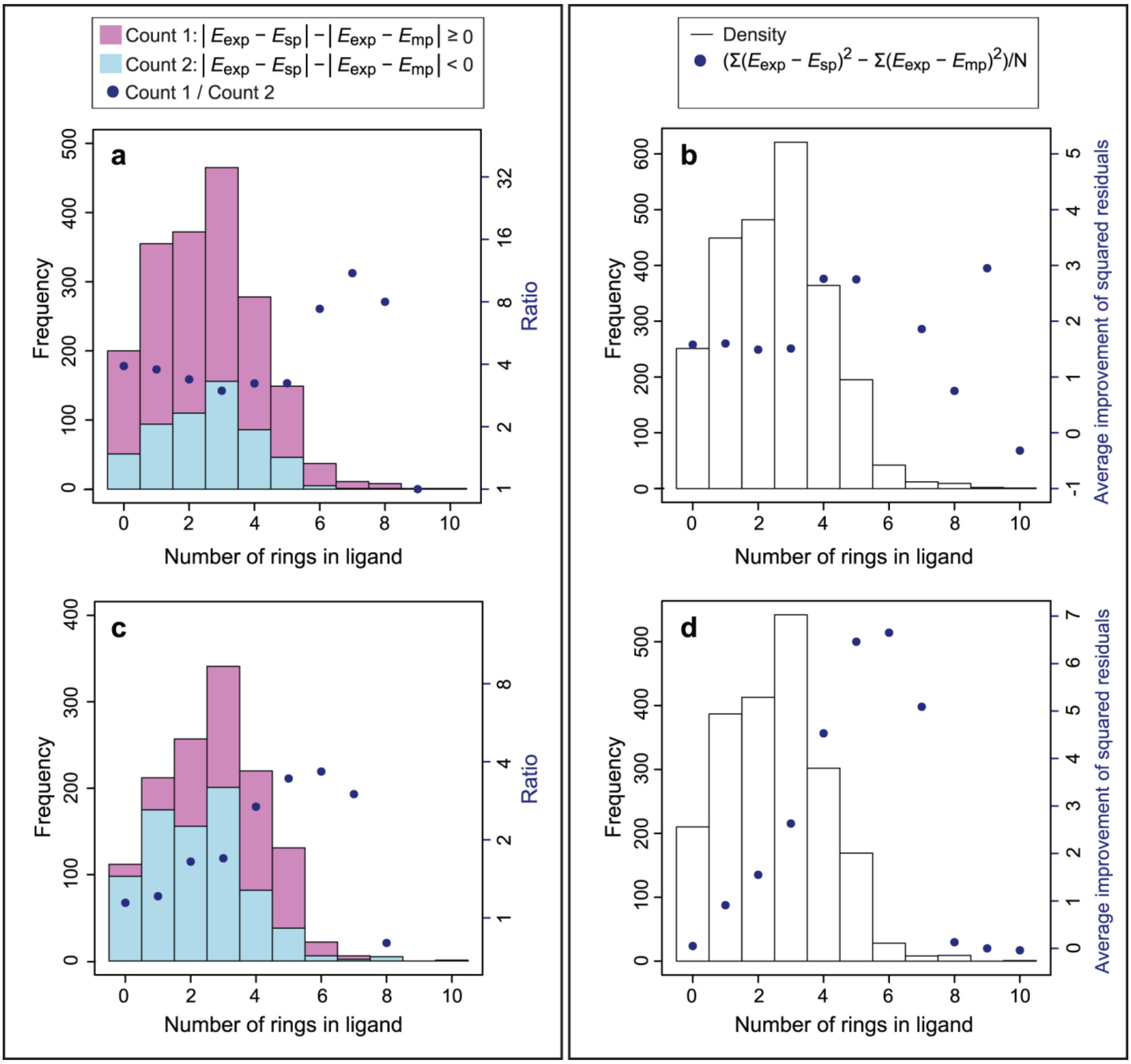

Figure S8. Analysis of the ligand charge in relation to the effect of multipose binding on binding affinity prediction for the two-pose case considering the top score and the best pose from the Refined-eHiTS (**a**,**b**) and the Refined-AutoDock combination (**c**,**d**). (**a**,**c**) Ratio of "improved" (pink) and "not-improved" (light blue) binding affinities; and (**b**,**d**) Histogram of the ligand property and average improvement of the squared residuals shown in dots.

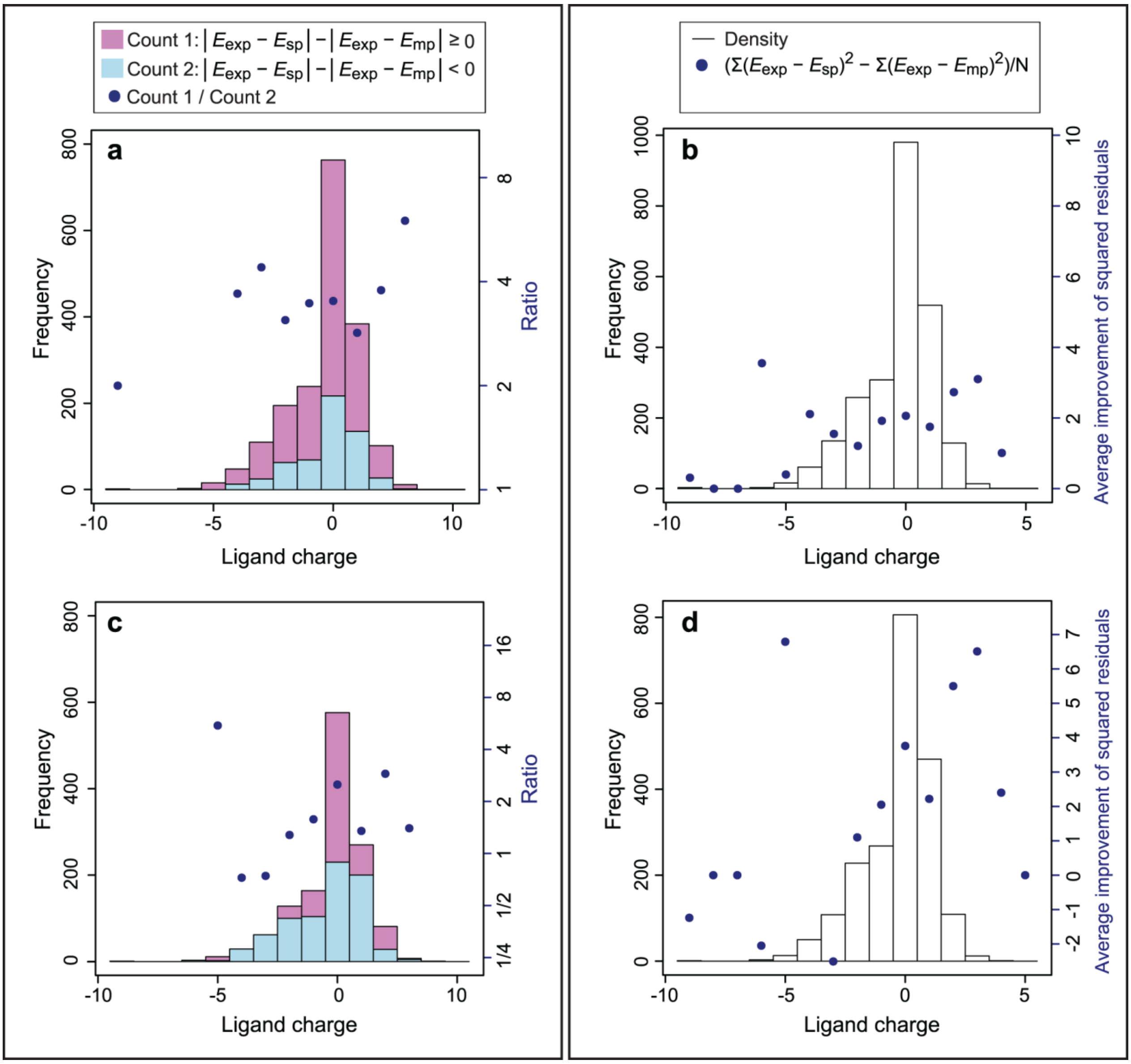

© 2014 by the authors; licensee MDPI, Basel, Switzerland. This article is an open access article distributed under the terms and conditions of the Creative Commons Attribution license (http://creativecommons.org/licenses/by/3.0/).
